# Food habit, physical activity and nutritional status of adolescents in selected schools of Madhyapur Thimi municipality, Nepal: A cross-sectional study

**DOI:** 10.1371/journal.pgph.0004136

**Published:** 2025-01-07

**Authors:** Samiksha Baral, Anil Kumar Singh, Arjun Aryal, Jwala Subedi, Utsav Dhakal, Anita Khanal

**Affiliations:** 1 Central Department of Public Health, Institute of Medicine, Tribhuvan University, Kathmandu, Nepal; 2 School of Public Health and Community Medicine, B. P. Koirala Institute of Health Sciences, Dharan, Nepal; 3 GoldenGate International, Tribhuvan University, Kathmandu, Nepal; Al-Bayan University, IRAQ

## Abstract

A dramatic rise in obesity is caused by unhealthy eating habits combined with lower levels of physical activity, and the under nutrition problem is still unresolved. Focusing on the nutritional needs of adolescents could be a significant step toward breaking the vicious cycle of malnutrition, chronic diseases, and poverty. This study aims to assess food habit, levels of physical activity and nutritional status of adolescents in Madhyapur Thimi Municipality, Bhaktapur. A cross-sectional descriptive study design was conducted. Cluster random sampling technique with validated, self-administered questionnaire was used for data collection. Ethical approval, written informed consent and assent were obtained. International Physical Activity Questionnaire (IPAQ) was used to measure level of physical activity. Nutritional Status was measured in terms of BMI for age z-score. Height and weight were measured by using UNICEF Stadiometer and Seca Scale. Data were analyzed using Chi-square test and logistic regression analysis was applied for the further analysis using SPSS V 20. Among the 460 participants, 19.6% were overweight, while 8.5% were underweight. Factors showing a statistical association with being overweight included the mother’s occupation, fruit avoidance, exposure to mass media advertisements, junk food consumption. Almost all the participants (93%) consumed junk food with (57.5%) consuming daily or alternately. Regarding physical activity, 65.4% of participants engaged in moderate activity, 30.7% were inactive and only 3.9% engaged in high levels of physical activity. The level of physical activity did not show a significant association with being overweight. Public health nutritionists should actively engage adolescents in developing interventions that promote healthy eating habits and reduce junk food consumption, as adolescents are more susceptible to food marketing than adults. Physical activity alone may not be responsible for being overweight as dietary modification plays vital role in maintaining normal body weight.

## Introduction

According to the World Health Organization (WHO), adolescents are those who are between the ages of 10-19 years old. Around 16% of the world’s population are adolescents and about 20% of them live in the South-East Asia region (SEAR) [1]. About 22% i.e. 6.38 million of Nepal’s total population are adolescents of 10–19 years’ age [2]. Physical, psychological, sexual, and social development shift quickly as people grow older and enter adulthood. Investing in the health of adolescent’s benefits them now, later in life, and for future generations’ health. Focusing on adolescent nutrition is essential for reducing risk factors for chronic diseases later in life [1].

Food habits are the how, what, and when an individual or group of individuals eat. Maintaining a balanced diet is crucial to ensure that the body receives the necessary nutrients to function optimally during this period of change. Individuals become more independent during adolescence and make many food choices on their own [3]. Healthy food consumption can help them satisfy their growing needs and enhance their academic performance [4]. During adolescence, being underweight, overweight, or obese can have detrimental impacts on one’s health [5]. Establishing healthy habits during adolescence is easier and more effective than trying to change harmful behaviors or treat health issues later in adulthood [6]. Low- and middle-income countries are actually going through a double burden of malnutrition. Children in these countries are more vulnerable to inadequate nutrition. At the same time, they are susceptible to high-sugar, high-fat, high-salt, micronutrient-poor, and energy-dense foods, which is affordable. This further contributes to their widespread consumption [7]. Adolescence is a nutritionally vulnerable time period. Adolescents have a lot to say about why they consume the foods they do, as well as perceptions of factors that could motivate them to make changes [8]. Nutritional effects in adolescent development extend beyond musculoskeletal growth, to neurodevelopment, cardiorespiratory fitness, and immunity [9]. Multi-Sector Nutrition Plan II (2018–-2022) is directly related to adolescent nutrition, whose objective is to improve adolescent nutrition by scaling up important nutrition-specific and sensitive interventions and establishing an enabling environment for nutrition [10].

In 2016, there were 50 million girls and 74 million boys worldwide who were obese and 75 million girls and 117 million boys worldwide who were underweight [5].Adolescents benefit from regular exercise in terms of their health and fitness. School-aged children can benefit from physical activity for their brain health including enhanced cognition (such as academic performance, memory, etc.) [11]. It’s likely that physical exercise habit formed during adolescence will last into adulthood. Overweight, obesity, and chronic disorders like diabetes, hypertension, cardiovascular diseases, and many types of cancer are all linked to sedentary behavior and lower levels of physical activity (i.e., those not meeting WHO standards) [12].

Focusing on the nutritional needs of adolescents could be a significant step toward breaking the vicious cycle of malnutrition, chronic diseases, and poverty [13]. Despite this fact, there are no adolescent-specific nutrition targets in Sustainable Development Goals (SDG) and WHO’s global action plan and even donor funding for adolescent nutrition is insufficient [14]. Global and national policy guidelines need to be reinforced with better data to support adolescents nutrition [15]. There is inadequate evidence on the factors that influence body weight and food habits among Nepalese adolescents. Since data on food habits is critical, this study is needed for a thorough understanding of the rising body weight problem in order to develop more effective ways for promoting healthy weight and food habits in adolescents. Findings from this study can help public health experts who work with adolescents to plan future interventions. This study will also help school administrators to develop healthy meal recommendations and provide healthy food to help minimize our country’s double burden of malnutrition. This study aims to assess food habit, level of physical activity and nutritional status of school-going adolescents in Madhyapur Thimi Municipality, Bhaktapur. Further aims were to assess the association of food habits and physical activity with overweight.

## Materials and methods

### Study design and method

Cross – sectional descriptive study design was used in the study with quantitative research method. Data collection took place from 20^th^ Dec, 2022 to 8^th^ of Feb, 2023.

### Study site

The study site was Madhyapur Thimi Municipality, Bhaktapur district of Bagmati Province, Nepal. The municipality covers an area of 11 square kilometers. Madhyapur Thimi is divided into 9 wards. According to the Central Bureau of Statistics (CBS) 2021 Census, the population of Madhyapur Thimi Municipality is 119,756 people. Madhyapur Thimi is one of Nepal’s ancient, cultural, and historical sites. It is one of the rapidly growing urban districts, with a recent increase in urban population [16]. Madhyapur Thimi Municipality, which was historically famed for its diverse farming and secure food supply, is rapidly transforming into a place that relies on food imports to meet food demand [17]. Ward no. 4 is selected as this ward is located at an urban setting, has better infrastructure viz. higher number of schools, better transportation facilities etc as compared to other wards [18]. Resultantly, students enrolled in this ward’s schools represent a microcosm of adolescents of the municipality. Similar study has not been conducted in this Municipality.

### Study population and unit

The study population were adolescents who were enrolled in secondary schools of grade 11 and 12 in ward no. 4 of Madhyapur Thimi Municipality. Study unit were individual adolescent students. Inclusion criteria were adolescent from the age group 16–19 years. As they are considered late adolescent by WHO [19]. Exclusion criteria were those who were not eligible to administer IPAQ and/or other tools.

### Sampling techniques

Cluster random sampling technique was used. As per the data received from Central for Education and Human Resource Development (CEHRD), there were 11 secondary schools in Madhyapur Thimi Municipality running grade 11 and 12. For the purpose of this study, all schools were selected from ward no. 4 based on above justification while grade 11 and 12 were taken as clusters. A proportionate sampling method was used to select 460 students from the total population across all schools, ensuring that the sample size from each school was proportional to its total student population. Selection of class was done applying random sampling. All students were included from selected cluster of grade 11 and 12.

### Sample size

The sample size for the study is calculated by using Cochran’s formula,


$$\text{Sample size }\left(\text{n}\right)=\frac{{\text{z}}^{\text{2}}\text{pq}}{{\text{d}}^{2}}$$

where, z = Standard normal deviate, usually set at 1.96 which correspond to 95% Confidence level.

p = Proportion of target population estimated to have a particular characteristic. Sample size estimated from prevalence of overweight was higher compared to underweight. So we take prevalence of overweight among adolescents from Singh et al., 2021 [20]; p = 0.237 = 23.7% q=0.763.

d = Degree of accuracy required, usually set at 0.05 or 5%.

hence,


$$\begin{array}{c} n=\;\frac{{\left(1.96\right)}^{2}\times \;0.237\;\times 0.763}{{\left(0.05\right)}^{2}}\\ =278 \end{array}$$

Taking 10% of the sample size as non- response rate, the final sample size will be 278 + 10% of 278= 306. Using design effect of 1.5, the sample size will be 306 × 1.5 = 459. We take the sample size (n) = 460 for convenience.

### Data collection tools and techniques

A self-administered questionnaire was used for data collection. The structured questionnaire adapted from the Nepalese Adolescent Nutrition Survey (2014), and Global School based health survey Nepal (2015) was used in the study; which is already a piloted study instrument. International Physical Activity Questionnaire (IPAQ) was used to measure physical activity [21]. Nutritional Status was measured in terms of BMI for age z-score. Seca Scale model number 874 was used to measure weight. UNICEF Stadiometer was used to measure height. Standard tools were provided by New ERA (Non-government organization) to measure height and weight. Data was collected using the self-administered questionnaire from participants who provided written informed consent (18 years and above) or assent (below 18 years) along with parents’ written informed consent to participate during their school day, and a 30–40-minute time was allocated to complete the questionnaire.

### List of variables

#### Dependent variables.

*Nutritional status (Measured in terms of BMI for age z-score):* It was measured in terms of Body mass index (BMI). BMI-for-age based on the WHO child and adolescent growth standards [22]. It is defined as a person’s weight in kilograms divided by the square of his height in meters (kg/m^2^). Study participants was classified as underweight (<−2SD from median for BMI by age and sex) and overweight (>+1SD from median for BMI by age and sex) [22] based on BMI Z-score.

#### Independent variables.

*Socio-demographic characteristics:* (Age, Sex, Religion, Parents education, Type of family, Family size, Parents occupation).

*Physical activity (Measured through IPAQ):* Physical activity were categorized as low, moderate (half an hour of at least moderate intensity activity on most days) or high activity levels (one hour of activity per day or more of at least moderate intensity activity level) [21].

**Food habit:** It comprises food frequency, and individual food choices, hunger, main diet, vegetarian/ non vegetarian, meat, fruit, vegetables, soft drink, and fast food consumption, Salty food consumption, High fat food consumption, Junk or processed food consumption, Skipping meal, Dairy.

**Adolescent:** For this study students studying in grades 11 and 12 between the age group 16–19 were considered to be school going adolescents [19].

### Validity and reliability of the study

#### Validity.

The set of questionnaire was checked and verified under close guidance of supervisor. Simple and understanding language was used as far as possible. Content validity was ensured by developing the questionnaire according to the objectives of the study and study variables. Face validity was maintained by translating the questionnaire into Nepali version. The pretested questionnaire used in Nepalese Adolescent Nutrition Survey (2014), and global School based health survey (2015) was used in the study. IPAQ has been validated and used in Nepal for measuring physical activity [21].

Seca Scale was calibrated regularly to assure that they are measuring correctly and accurately. The weighing scale was placed at a horizontal level and indication was zeroed before the test. Well known weight was measured again to ensure it works normally. Adolescents were requested to open their shoes, sweater and ornaments and made to stand straight and still on the foot step of Seca Scale. Adolescents were given some time to stand still until the measurement was constant. Measurement was recorded instantly by standing in front of the Seca Scale.

For measuring height, height board was placed in a horizontal place and its back was supported. Adolescents were requested to open their shoes, socks, cap and other head ornaments and to stand on the height board touching their back. While standing; head, shoulder, back legs and heels touched the board, knees were straight and soles were joined. Both hands were straight facing downward. Head was straight and adolescent faced front. Eyes and ears were parallel to the ground. Measurement was done by two persons. One worked on adjusting the legs and other worked on head. Person holding the head read the scale of the board.

#### Reliability.

Pre-testing was done and modification of instrument was done after pre-testing. It was done in 10% i.e. 40 students by randomly selecting one school of Madhyapur Thimi Municipality. To ensure interrater reliability, measurements were compared, and any discrepancies were resolved through repeated measurements to ensure consistency.

### Ethical consideration

Formal approval was taken from Institutional Review Committee (IRC) of Institute of medicine (IOM), Tribhuvan University (Ref:285(6-11) E2). Permission was taken from Municipality Office and respective school. Written informed consent was taken from the parents whose children were below 18 years before data collection and objectives of research were clarified. Written informed consent was taken from adolescents who were 18 years and above. Written informed assent was taken from adolescents who were below 18 years. No pressure or inducement of any kind was given to the subjects. Utilization of collected information was for the study purpose. Participants were informed about their nutritional status so that necessary steps could be taken for prevention.

### Data management and analysis

The data was coded, entered, analyzed and interpreted according to the objective of the study using computer software Statistical Package for Social Sciences (SPSS V 20). Descriptive results were presented in the form of mean, standard deviation, frequency, and percentage. The association of different independent variables with dependent variables was determined by using Chi-square test and other relevant tests. Logistic regression analyses were applied for the further analysis. The adjusted relationship of independent variables with being overweight was assessed at 95% CI in the multivariate analysis. The adjustment of factors which could be possible confounders to the dependent variable was done with binary logistic regression using enter method. Seven variables that were found significant at 95% level of significance in bivariate analyses were entered into multivariate analysis. Prior to inclusion of variables in the regression model, Variance inflation factor (VIF) was checked using collinearity diagnostics. No problems of collinearity among the independent variables were identified.

## Results

### Socio-demographic characteristics of the study population

Table 1 provides the description of socio-demographic characteristics of the study population. Out of 460 participants, the mean age of participants was 17.19 ± 0.883 years. Majority of participants (83.7%) were Hindu, and (78.3%) lived in a nuclear family. Majority of fathers (84.7%) of participants were employed but only 39.8% mothers of participants were employed.

**Table 1:** Socio-demographic characteristics of the adolescent (n = 460).

Study variable		Number	Percent
**Age in years** (mean ± SD) (17.19 ± 0.883)	16–17	298	64.8
	18–19	162	35.2
**Sex**	Male	243	52.8
	Female	217	47.2
**Religion**	Hindu	385	83.7
	Buddhism	42	9.1
	Christian	23	5
	Kirat	7	1.5
	Muslim	3	0.7
**Family type**	Nuclear	360	78.3
	Joint/Extended	100	21.7
**Family size** (mean ± SD) (5.26 ±2.017)	More than 4	260	56.5
	Less than or equal to 4	200	43.5
**Class**	Class 12	231	50.2
	Class 11	229	49.8
**Father’s education**	No education	55	12
	Basic level education	111	24.1
	Secondary level education	261	56.7
	More than secondary level	33	7.2
**Mother’s education**	No education	87	18.9
	Basic level education	174	37.8
	Secondary level education	183	39.8
	More than secondary level	16	3.5
**Father’s occupation**	Employed	390	84.7
	Unemployed	70	15.3
**Mother’s occupation**	Employed	183	39.8
	Unemployed	277	60.2

### Food habit of the study population

In Table 2 it is depicted that majority (71.7%) of the participants never went hungry. Almost all the participants (93.3%) had rice as a main diet. Large majority of participants (91.3) were non-vegetarian among them almost all participants (93.6%) had consumed meat more than or once a week. Majority of the participants (61.5%) had vegetables less than or 1 time per day. Majority of participants (61.7%) had fruits less than 1 time per day. Half of the participants (50.7%) had carbonated soft drink less than 1 time per day. Nearly half of the participants (49.8%) consumed fast-food less than or 2 days in a week. Most participants (62.2%) watched advertisements of carbonated soft drinks or fast food. Majority of participants (63.7%) were taught about the benefits of eating fruits and vegetables in school. Almost all the student (93%) consumed junk food where (57.5%) were found consuming daily or alternately.

**Table 2:** Food habits of the adolescent (n = 460).

Study variable		Number	Percent
**Hungry**	Never	330	71.7
	Yes	130	28.3
**Main diet**	Rice	429	93.3
	Wheat	26	5.7
	Maize	5	1.1
**Meal preference**	Non-vegetarian	420	91.3
	Vegetarian	40	8.7
**Meat consumption n = 420**	Once a week or more	393	93.6
	Not at all	27	6.4
**Vegetables consumption**	1 times per day or less	283	61.5
	≥2 times per day	177	38.5
**Fruits consumption**	<1 times per day	284	61.7
	1 times per day or more	176	38.3
**Carbonated soft drink consumption**	<1 time per day	233	50.7
	Not at all	147	32
	1 times per day or more	80	17.4
**Fast-food consumption**	≤2 days	229	49.8
	≥3 day	149	32.4
	Not at all	82	17.8
**Salty food consumption**	≥1 time per day	247	53.7
	Less than 1 time per day	213	46.3
**Fat food consumption**	Less than 1 time per day	262	57
	≥1 time per day	198	43
**Watched advertisements**	Yes	286	62.2
	No	174	37.8
**Taught the benefits of fruits and vegetables**	Yes	293	63.7
	No	167	36.3
**Junk food**	Yes	428	93
	No	32	7
**Junk food (**n = 428)	Daily/alternately	246	57.5
	≤ Twice a week	182	42.5
**Skipping any meal**	Yes	315	68.5
	No	145	31.5
**Skipped meal (**n = 315)	Dinner	122	38.7
	Breakfast	92	29.2
	Day snacks	63	20
	Lunch	38	12.1
**Main Reason for skipping meal** (n = 315)	No time to have meal	97	30.8
	No hunger	83	26.3
	Health conscious	67	21.3
	For weight loss	52	16.6
	For attractive figure	11	3.5
	Lack of food at house	5	1.6
**Dairy Consumption**	Less than 1 time per day	248	53.9
	≥1 time per day	212	46.1

### Physical activity level of the study participants

Table 3 shows majority of participants (65.4%) had moderate Physical activity level. About 30.7% of participants were inactive. Only 3.9% had high level of physical activity.

**Table 3:** Physical activity level of the adolescent n = 460.

Physical activity level	Number	Percent
Low PA	141	30.7
Moderate PA	301	65.4
High PA	18	3.9

### BMI of study participants

Table 4 shows that majority of participants (72%) had normal BMI while 19.6% were overweight and 8.5% were found to be underweight.

**Table 4:** BMI of the adolescent (n = 460).

BMI	Number	Percent
Underweight	39	8.5
Normal	331	72
Overweight	90	19.6

### Association of overweight with socio-demographic characteristics

Table 5 presents the association of socio-demographic factors with being overweight. The variable that showed statistical association with being overweight includes occupation of father (χ2 = 4.8, p-value 0.028), occupation of mother (χ2 = 10.04, p-value 0.002). The socio-demographic factors such as sex, age, religion, family size, parent’s education and grade did not show any significant association with being overweight/obese.

**Table 5:** Association of socio-demographic variables with overweight (n = 460).

Study variable	Overweight	Non-Overweight	Chi-square value (χ2)	p-value
**Age**				
16–17	56 (12.2%)	242 (52.6%)	0.3	0.571
18–19	34 (7.4%)	128 (27.8%)		
**Sex**				
Male	54 (11.7%)	189 (41.1%)	2.3	0.128
female	36 (7.8%)	181 (39.3%)		
**Religion**				
Hindu	73 (15.9%)	312 (67.8%)	0.5	0.459
Non-Hindu	17 (3.7%)	58 (12.6%)		
**Family type**				
Nuclear	70 (15.2%)	290 (63%)	0.01	0.901
Joint/Extended	20 (4.3%)	80 (17.4%)		
**Family size**				
≤4	36 (7.8%)	164 (35.7%)	0.5	0.458
>4	54 (11.7%)	206 (44.8%)		
**Fathers education**				
≤Basic level education	32 (7%)	134 (29.1%)	0.01	0.907
≥secondary level education	58 (12.6%)	236 (51.3%)		
**Mothers education**				
≤Basic level education	54 (11.7%)	207 (45%)	0.5	0.486
≥Secondary level education	36 (7.8%)	163 (35.4%)		
**Occupation of father**				
Employed	83 (18.1%)	306 (66.7%)	4.8	0.028*
Unemployed	7 (1.5%)	63 (13.7%)		
**Occupation of mother**				
Employed	49 (10.7%)	134 (29.1%)	10.04	0.002*
Unemployed	41 (8.9%)	236 (51.3%)		
**Grade**				
Grade 11	51 (11.1%)	178 (38.7%)	2.1	0.145
Grade 12	39 (8.5%)	192 (41.7%)		

* Statistically significant at 5% level of significance.

### Association of overweight with food habit

Table 6 presents the association of Food habit with being overweight. Factors that showed statistical association with being overweight include fruit consumption (χ2 = 29.4, p-value <0.001), Fast food consumption (χ2 = 4.2, p-value 0.041), watched advertisement for carbonated soft drinks or fast food (χ2 = 9.993, p-value 0.002), junk food consumption (χ2 =12.998, p-value <0.001) and reason for skipping meal (χ2 = 5.96, p-value 0.015). Other food habits did not show any significant association with being overweight.

**Table 6:** Association of overweight/obesity with food habit (n = 460).

Study Variable	Overweight	Non- Overweight	Chi-square Value (χ2)	P-value
**Hungry**				
Never	65(14.1%)	265(57.6%)	0.013	0.910
yes	25 (5.4%)	105 (22.8%)		
**Main diet**				
Rice	86 (18.7%)	343 (74.6%)	0.5	0.463
Others (wheat and maize)	4 (0.9%)	27 (5.9%)		
**Meal preference**				
Vegetarian	6 (1.3%)	34 (7.4%)	0.6	0.446
Non-vegetarian	84 (18.3%)	336 (73%)		
**Meat consumption**				
Not consumed at all	3 (0.7%)	24 (5.7%)	0.9	0.345
Once a week	81 (19.3%)	312 (74.3%)		
**Vegetable consumption**				
≤1 times per day	61 (13.3%)	222 (48.3%)	1.8	0.174
≥2 times per day	29 (6.3%)	148 (32.2%)		
**Fruits consumption**				
<1 times per day	78 (17%)	206 (44.8%)	29.4	<0.001
≥1 times per day	12 (2.6%)	164 (35.7%)		
**Carbonated soft drink consumption**				
<1 times per day	69 (15%)	311 (67.6%)	2.7	0.097
≥1 times per day	21 (4.6%)	59 (12.8%)		
**Fast- food from restaurant**				
≤2day	69 (15%)	242 (52.6%)	4.2	0.041*
≥3 times per day	21 (4.6%)	128 (27.8%)		
**Salty food consumption**				
<1 times per day	40 (8.7%)	173 (37.6%)	0.2	0.693
≥1 times per day	50 (10.9%)	197 (42.8%)		
**High fat food consumption**				
<1 times per day	47 (10.2%)	215 (46.7%)	1.0	0.312
≥1 times per day	43 (9.3%)	155 (33.7%)		
**Watched advertisement for carbonated soft drinks or fast food**				
Yes	21 (4.6%)	153 (33.3%)	9.9	0.002*
No	69 (15%)	217 (47.2%)		
**Taught benefits of eating more fruits and vegetable**				
Yes	54 (11.7%)	239 (52%)	0.7	0.416
No	36 (7.8%)	131 (28.5%)		
**Junk food consumption**				
Yes	81 (17.6%)	347 (75.4%)	1.6	0.206
No	9 (2%)	23 (5%)		
**Junk food Consumption**				
Daily/alternately	61 (14.3%)	185 (43.2%)	12.9	<0.001*
≤Twice a week	20 (4.7%)	162 (37.9%)		
**Skipping any meal**				
Yes	63 (13.7%)	253 (55%)	0.09	0.766
No	27 (5.9%)	117 (25.4%)		
**Skipped meal**				
Breakfast/ Day snacks	30 (9.5%)	125 (39.7%)	0.2	0.676
Lunch/Dinner	34 (10.8%)	126 (40%))		
**Reason for skipping**				
Health conscious	35 (11.1%)	95 (30.2%)	5.9	0.015*
Others	29 (9.2%)	156 (49.5)		
**Dairy consumption**				
<1 times per day	43 (9.3%)	205 (44.6%)	1.7	0.193
≥1 times per day	47 (10.2%)	165 (35.9%)		

* Statistically significant at 5% level of significance.

### Association of overweight with physical activity

Table 7 presents the association of level of physical activity with being overweight. Physical activity did not show any significant association with overweight.

**Table 7:** Association of overweight/obesity with physical activity (n = 460).

Physical Activity Level	Overweight	Non- Overweight	Chi-square	P-value
Low PA	28 (6.1%)	113 (24.6%)	0.011	0.916
Moderate/high PA	62 (13.5%)	257 (55.9%)		

* Statistically significant at 5% level of significance.

### Adjusted relationship of independent variables with overweight

Table 8 shows the unadjusted and adjusted ORs and their 95% CIs of factors associated with being overweight. Results from regression analysis showed mother’s occupation, fruit avoidance, fast food consumption from restaurant, exposure to advertisement for carbonated soft drinks and junk food consumption as having significant association with being overweight.

**Table 8:** Adjusted relationship of independent variables with overweight (n = 460).

Study variables	COR	95% CI	AOR	95%CI	p-value
**Occupation of father**					
Employed	2.4	1.1–5.5	2.3	0.7–7.4	0.150
Unemployed	Ref.		Ref.		
**Occupation of mother**					
Employed	2.1	1.3–3.4	3.08	1.6–6.0	**0.001** *
Unemployed	Ref.		Ref.		
**Fruits consumption**					
<1 times per day	5.2	2.7–9.8	5.7	2.5–13.3	**<0.001** *
≥1 times per day	Ref.		Ref.		
**Fast-food from restaurant**					
≤2 day	1.7	1.09–2.9	1.1	0.5–2.2	0.829
≥3 times per day	Ref.	Ref.	Ref.		
**Watched advertisement for carbonated soft drinks or fast food**					
No	0.4	0.2–0.7	0.3	0.1–0.6	**0.002** *
Yes	Ref.		Ref.		
**Junk food Consumption**					
Daily/alternately	2.7	1.5–4.6	3.1	1.5–6.6	**0.003** *
≤Twice a week	Ref.		Ref.		
**Reason for skipping**					
Health conscious	1.9	1.1–3.4	0.5	0.3–1.0	0.056
Others	Ref.		Ref.		

* Statistically significant at 5% level of significance.

Results shows that adolescents whose mother were employed were 3.08 times more likely to be overweight compared to unemployed mothers (AOR 3.08, 95% CI 1.6–6). Adolescents who consumed fruits less than 1 time per day were 5.7 times more likely to be overweight compared to those who consume more than or one time per day (AOR 5.7, 95% CI (2.5–13.3). Participants who did not watch advertisement of carbonated soft drinks or fast food were 0.3 times less likely of being overweight (AOR 0.3, 95% CI 0.1–0.6) compared to those who watched. Participants who consumed junk food daily/alternately were 3.1 times more likely to be overweight compared to less than or twice a week (AOR 3.1 95%, CI 1.5–6.6).

## Discussion

Out of 460 adolescents, 19.6% were overweight and 8.5% were underweight. Study in 2022 by Singh et al., showe` vd 9.1% underweight and 23.7% overweight which is similar to this study [20]. Results were also consistent with WHO fact sheet [12]. Both the studies used WHO BMI for age classification for measuring nutritional status of adolescents [22]. The NDHS 2022 found that among adolescent females, 26% were underweight and 6% were overweight which differs from this study [23]. Another study conducted in Kathmandu Valley contradict from this study which shows 27.8% underweight and 5% overweight. These difference might be because the study was conducted in public schools, where most students may come from lower economic backgrounds, and the BMI classification for adults was used to measure nutritional status in those studies [24]. Previous studies showed that (29.9–27.4)% adolescent were underweight and (7.6–8)% were overweight respectively [25]. In a cross-sectional survey of urban school adolescents in Nepal, 12.2% of teenage students were overweight [26].These studies show a significant low prevalence of overweight as compared to this study. The classification of BMI was not clearly mentioned in those studies.

This study indicates that prevalence of thinness had slightly decreased, whereas prevalence of overweight had increased in compared to the previous studies. According to the DHS 2018 Adolescent Nutrition Report, in most nations, overweight and obesity were a greater problem than thinness, which is consistent with this study finding. However there are differences between East Asia and the Pacific where the prevalence of thinness is the same as that of overweight and obesity, and South Asia where the prevalence of thinness is double that of overweight and obesity [15]. However, as compared to prior investigations, this study found that the prevalence of thinness had somewhat decreased, while the prevalence of overweight had increased. Overweight and obesity, once thought to be an issue only in high-income nations, are increasingly becoming more prevalent in low- and middle-income nations, especially in urban areas. In 2016, there were more than 340 million overweight children and adolescents between the age 5 to 19. From 4% in 1975 to over 18% in 2016 WHO, the prevalence of overweight among children and adolescents aged 5-19 has increased considerably [7]. According to the WHO European Region, children who are overweight have a higher likelihood of becoming overweight as adults and developing non-communicable diseases at an earlier age. One out of every three children in the WHO European Region is overweight or obese. Over 60% of children who gain weight before puberty will also gain weight as adults [27].

This study showed significant association of overweight with occupation of mother. Consistent to this findings, Anderson et al. discovered that a child is more likely to be overweight if his or her mother worked more hours per week [28]. Similar findings were obtained in a study conducted in the United Kingdom [29] and study in low and middle income countries [30]. Study in Kathmandu also reported adolescents whose mothers worked had a higher likelihood of being overweight [20].This could be because mothers do not have enough time to prepare nutritious meals for their children. Furthermore, adolescent may have consumed high-calorie foods away from home. Long work hours for mothers have an impact on the overweight status of their children [31].

In this study, adolescents who did not consumed fruits at least one time per day were found to have higher risks of being overweight. More than half of adolescents (54.3%) did not consume fruits at least 1 time per day which is similar to the study conducted in Nepal [20]. Findings are consistent with a South Asian study that found nutritional risk factors related with overweight/obesity included decreasing fruit consumption and increasing fast food consumption [32]. According to 2014 Nepal Adolescent Nutrition Survey, fruits and vegetables appear to provide protective effects against malnutrition [33]. Study conducted in Finnish adolescent in 2019 [34] and a cross-sectional survey of urban school adolescents in Nepal, also had similar results [26]. The role of fruits in preventing overweight and obesity is connected to its low calorie density, high dietary fiber content, and associated enhancing satiety effect [35]. According to the Steps survey 2019, eating fewer than five servings of fruit and vegetables per day was the main risk factor for NCDs [36]. Adolescents should consume fruits and vegetables minimum 400 g/day advised by the WHO and the Food and Agriculture Organization (FAO) in several nations, including Europe [37].

Less participants were taught about the benefits of eating more fruits and vegetables in school. Although the National Health Policy 2076 emphasizes to develop and conduct the school health program as well as provide nutritional education [38]. The purpose of the school health and nutrition strategy 2006 is to improve students’ health and nutrition behavior and habits, and the strategy states that life skills-based health education is essential for attaining and maintaining behavioral change [39].

Most student (93%) consumed junk food in this study which is consistent with the findings from Singh et al., [20], adolescent nutrition survey 2014 [33] and study from Pokhara in 2021 [40]. Many adolescents value food convenience, and they will consume an excessive amount of the inappropriate types of food, such as fast food, soft drinks, or processed foods [3]. In this study, adolescents who consumed junk food daily/alternately were found to have higher risks of being overweight. Participants who consumed junk food daily/alternately were 3.1 times more likely to be overweight, this findings were supported by study conducted in Pokhara [40] and another study from Pokhara in 2017 [41]. Similar study in Butwal reported that late adolescents in urban areas were unable to maintain a normal BMI due to their unhealthy eating habits [42]. Adolescents who had a habit of junk food consumptions were at elevated risk of being overweight [20]. Global environmental and societal changes related to development and a lack of supportive policies in areas like health, agriculture, food processing, transport, education, marketing, environment, distribution, and city planning result in increased consumption of energy-dense meals that are excessively high in fat and sugars [7].

Participants who did not watch advertisement of carbonated soft drinks or fast food were protective against overweight. This is supported by a study conducted in Pokhara in 2017, where the majority of adolescents (82.4%) consumed food products advertised in mass media [41].Similar pattern were observed from study in Chitwan [43]. Advertisement influence food choices of the adolescents. Governments and concerned regulatory authorities should control the mass media from advertising unhealthy junk foods.

Majority of participants (65.4%) had moderate level of physical activity and only 3.9% had high level of physical activity. Besides 30.7% of participants were inactive. There was no significant association between physical activity and nutritional status in this study which differs from the study conducted in Kathmandu, Nepal that highlighted overweight/obesity was more common among those adolescent who were physically inactive [20]. Another study conducted in public school of Kathmandu Valley, Nepal reveals the same findings [24]. In contrast to this study findings, data from the 2015 National Health and Morbidity study (NHMS), a countrywide cross-sectional study, suggest that a low level of PA is linked with a higher risk of overweight/obesity than a high level of PA [44]. According to a study on Syrian adolescents, the prevalence of overweight/obesity has a strong inverse relationship with physical activity [45]. Several studies have found a link between a lack of physical activity and being overweight or obese [46,47].This difference might be because of different tools used to assess physical activity level. This could be because one week of physical activity will not change the nutritional status of adolescent, it must be continuous effort at least for a month or a year. Physical exercise alone is not responsible for becoming overweight or obese, as dietary changes play an important part in maintaining a normal body weight.

## Conclusions and recommendation

### Conclusion

The findings show that overweight is more prevalent than underweight. We can see the transition that prevalence of thinness had slightly decreased, whereas prevalence of overweight had increased in compared to the previous studies. Junk food consumption, mass media advertisement, fruit avoidance, and mothers’ occupation status were all key contributors for being overweight among adolescents. Adolescents who consumed junk food daily/alternately were found to have higher risks of being overweight. Participants who consumed fruits and who did not watch advertisement of carbonated soft drinks or fast food were less likely to be overweight. More than half were involved in moderate level of physical activity whereas fewer participants were found to have high level of Physical activity. There was no significant association between physical activity and being overweight in this study. Physical activity alone may not be responsible for being overweight as dietary modification plays vital role in maintaining normal body weight.

### Recommendation

Periodically assess adolescents’ BMI status, nutrition data and health behaviors in order to adjust their food habits and other lifestyle behaviors on time to support their growth and development. Governments and concerned regulatory authorities should control the mass media from advertising unhealthy junk foods and adopt long-term realistic health-promoting policies. The school and community should conduct an awareness campaign about junk food intake and its negative consequences and create a suitable environment at school and at home. Adolescents should get prompt interventions and adolescent nutrition advocacy must be done to enhance lifestyle behavior and minimize overweight. Public health nutritionists should engage adolescents actively in creating interventions that promote healthy eating habits and reduce the consumption of junk food because adolescents are more susceptible to food marketing than adults. More information on micronutrient status and food quantity is recommended for future research.

### Limitation and generalizability of the study

Recall bias may have occurred as adolescent had to remember frequency of their food consumption in the last 7 days. Since the tool was self-administered questionnaire, some participants may interpret the question differently so there may be information bias. It was minimized by explaining the participants about the tools in detail and enough time was given to complete the questionnaire.

The study provides valuable insights into the nutritional and physical activity patterns of adolescents in Madhyapur Thimi Municipality, with potential relevance for similar urban populations in Nepal. This study uses a cluster random sampling technique to select 460 school-going adolescents, which is generally a robust method for ensuring that the sample is representative of the target population within Madhyapur Thimi Municipality. The sample size (460 participants) is relatively large and may provide sufficient statistical power to detect associations between variables. The study uses validated tools such as the International Physical Activity Questionnaire (IPAQ) for measuring physical activity and standard equipment (UNICEF Stadiometer and Seca Scale) for assessing height and weight. This enhances the internal validity of the findings and suggests that similar results may be obtained if the study is replicated using the same methods. The BMI-for-age z-score is a widely accepted measure of nutritional status in adolescents, making the findings comparable to other studies using the same metric.

The findings may not be generalizable to adolescents in other regions of Nepal, especially rural areas. The sample includes only school-going adolescents, excluding those who are out of school. Adolescents who do not attend school may have different nutritional and physical activity patterns, potentially limiting the applicability of the findings to the broader adolescent population. Future research involving diverse geographic regions, both urban and rural settings, and longitudinal designs could improve the generalizability and applicability of the results to national or international contexts.

## Supporting information

S1 Questionnaire Study questionnaire. (SAV)

S1 Dataset Study dataset. (DOCX)
