# Supplementary material for: Food habit, physical activity and nutritional status of adolescents in selected schools of Madhyapur Thimi municipality, Nepal: A cross-sectional study
Source: PLOS Glob Public Health. 2025 Jan 7;5(1):e0004136. doi: 10.1371/journal.pgph.0004136 (PMC11706376; doi:10.1371/journal.pgph.0004136)
Supplement: S1 Dataset — (DOCX) [file pgph.0004136.s002.docx]

| Date ============================================ | | Roll No. =============================== | | |
| --- | --- | --- | --- | --- |
| **Anthropometric measurement**  **Weight(Kg)]**================Kg & **Height(cm)** ===================cm | | | | |
| **Q.N** | **Questions** | | **Responses** | |
| **1** | **Socio-Demographic Information** | | | |
| 1.1 | Age  Date of Birth | | ……………….  ………..Year /…….Month / ….. Day | |
| 1.2 | Sex | | a. Female b. Male | |
| 1.3 | Religion | | a. Hindu b. Buddhism  c. Muslim d. Kirat  e. Christian f. Others ============== | |
| 1.4 | Type of family | | a. Nuclear b. Joint  c. Extended | |
| 1.5 | Family size | | =========================================== | |
| 1.6 | Father’s Education | | ……………………… Class | |
| 1.7 | Mother’s Education | | ……………………… Class­ | |
| 1.8 | Occupation of father | | a. Government Office  b. NGO / Private  c. Agriculture  d. Business  e. Foreign employment  f. Daily Labor  g. Retired  h. Unemployed  i. Household worker  j. Other…………….. | |
| 1.9 | Occupation of mother | | a. Government Office  b. NGO / Private  c. Agriculture  d. Business  e. Foreign employment  f. Daily Labor  g. Retired  h. Unemployed  i. Housewife  j. Other …………….. | |
| 1.10 | Number of earning members in the family | | a. One b. Two  c. More than 2 c. None | |
| 1.11 | Which class/Grade are you studying now? | | a. class 11  b. class 12 | |
| 2 | **Food Habit** | | | |
| 2.1 | During the past 7 days, how often did you go hungry because there was not enough food in your home? | | a. Never b. Rarely  c. Sometimes d. Most of the time  e. Always | |
| 2.2 | What is your main diet at home? | | a. Rice/daal b. Wheat  c. Maize d. Millet  e. Buckwheat f. Barley  g. Others …………………. | |
| 2.3 | Are you vegetarian/non-vegetarian? | | a. Vegetarian If vegetarian go to QN 2.5  b. Non-vegetarian  c. Eggitarian  d. Others (Specify) …………………. | |
| 2.4 | If non vegetarian, how often do you eat meat? | | a. Daily  b. Alternately  c. Once a week  d. Twice a week  e. Once a month  f. Twice a month  g. Others (Specify) …………………. | |
| 2.5 | During last 7 days, how many times per days did you eat Vegetables? Cauliflower, cabbage, or Brinjal etc. | | a. I did not eat vegetables during the past 7days  b. Less than one time per day  c. 1 time per day  d. 2 times per day  e. 3 times per day  f. 4 times per day  g. 5 or more times per day | |
| 2.6 | During last 7 days, how many times per days did you eat fruits? such as apples, oranges, mangoes, or papayas | | a. I did not eat fruit during the past 7 days  b. Less than one time per day  c. 1 time per day  d. 2 times per day  e. 3 times per day  f. 4 times per day  g. 5 or more times per day | |
| 2.7 | During the past 7 days, how many times per day did you usually drink carbonated soft drinks, such as Coca-Cola, Pepsi, or Sprite? (Do not include diet soft drinks.) | | a. I did not drink carbonated soft drinks during the past 7 days  b. Less than one time per day  c. 1 time per day  d. 2 times per day  e. 3 times per day  f. 4 times per day  g. 5 or more times per day | |
| 2.8 | During the past 7 days, on how many days did you eat food from a fast-food restaurant, such as mo mo, chowmein, burgers, or pizza? | | a. 0 days b. 1 day  c. 2 days d. 3 days  e. 4 days f. 5 days  g. 6 days h. 7 days | |
| 2.9 | During the past 7 days, how many times per day did you usually eat salty foods, such as noodles, crackers, daalmoth (mixtures), or paapad? | | a. I did not eat salty foods  b. Less than 1 time per day  c. 1 time per day  d. 2 times per day  e. 3 times per day  f. 4 times per day  g. 5 or more times per day | |
| 2.10 | During the past 7 days, how many times per day did you usually eat food high in fat, such as ghee, fried food, ice cream, or cream doughnuts? | | a. I did not eat food high in fat  b. Less than 1 time per day  c. 1 time per day  d. 2 times per day  e. 3 times per day  f. 4 times per day  g. 5 or more times per day | |
| 2.11 | During the past 7 days, how many advertisements for carbonated soft drinks or fast food did you see when you watched television/social media? | | a. I did not watch at all  b. A lot  c. A few  d. None | |
| 2.12 | During this school year, were you taught in any of your classes the benefits of eating more fruits and vegetables? | | a. Yes  b. No  c. I do not know | |
| 2.13 | Do you consume junk food or processed food? (such as Chips, Kurkure, Lays, Noodles, Salty Cookies, Biscuits, Cakes) | | a. Yes  b. No If no go to QN 2.15 | |
| 2.14 | If yes how often? | | a. Daily  b. Alternately  c. Once a week  d. Twice a week  e. Once a month  f. Twice a month  g. Others (Specify) …………………. | |
| 2.15 | How many times do you consume food in a day? | | …………………….…… times | |
| 2.16 | In a typical week, are you skipping any meal? | | a. Yes  b. No If no go to QN.2.19 | |
| 2.17 | If yes, what you skip? | | a. Breakfast b. Lunch  c. Day snacks d. Dinner  e. Others (Specify) …………………. | |
| 2.18 | What is the main reason for skipping meal? | | a. Lack of Food at House  b. For weight loss  c. For attractive figure  d. Health conscious  e. No time to have meal  f. Others (Specify) …………………. | |
| 2.19 | During last 7days, how many times per days did you eat Dairy (Milk and milk product things) (Yoghurt, Cheese, paneer etc)? | | a. I did not eat Dairy during the past 7 days  b. Less than one time per day  c. 1 time per day  d. 2 times per day  e. 3 times per day  f. 4 times per day  g. 5 or more times per day | |
| 3 | **International Physical activity questionnaire (August 2002)** | | | |
| 3.1 | During the last 7 days, how many days did you do vigorous physical activities like heavy lifting, digging, aerobics, or fast bicycling? | | | a. _____ days per week  b. No vigorous physical activities -Skip to question 3.3 |
| 3.2 | How much times did you usually spend doing vigorous physical activities on one of those days? | | | a._____ hours per day  b._____ minutes per day  c. Don’t know/Not sure |
| 3.3 | During the last 7 days, how many days did you do moderate physical activities like carrying light loads, bicycling at a regular pace, or play doubles tennis? Do not include walking. | | | a._____ days per week  b. No moderate physical activities- Skip to question 3.5 |
| 3.4 | How much time did you usually spend doing moderate physical activities on one of those days? | | | a._____ hours per day  b._____ minutes per day  c. Don’t know/Not sure |
| 3.5 | During the last 7 days, how many days did you walk for at least 10 minutes at a time? | | | a._____ days per week  b. No walking- Skip to question 3.7 |
| 3.6 | How much time did you usually spend walking on one of those days? | | | a._____ hours per day  b._____ minutes per day  c. Don’t know/Not sure |
| 3.7 | During the last 7 days, how much time did you spend sitting in a stationary mode? | | | a._____ hours per day  b._____ minutes per day  c. Don’t know/Not sure |

**Thank you for Participating**
